# Ion-Based Cellular Signal Transmission, Principles of Minimum Information Loss, and Evolution by Natural Selection

**DOI:** 10.3390/ijms21010009

**Published:** 2019-12-18

**Authors:** B. Roy Frieden, Robert Gatenby

**Affiliations:** 1 College of Optical Sciences, University of Arizona, Tucson, AZ 85721, USA; friedenr@optics.arizona.edu; 2 Departments of Radiology and Integrated Mathematical Oncology, Moffitt Cancer Center, 1292 Magnolia Drive, Tampa, FL 33612, USA

**Keywords:** information, signal transmission, evolution, extreme physical information

## Abstract

The Extreme Physical Information EPI principle states that maximum information transmission or, equivalently, a minimum information loss is a fundamental property of nature. Prior work has demonstrated the universal EPI principle allows derivation of nearly all physical laws. Here, we investigate whether EPI can similarly give rise to the fundamental law of life: Evolution. Living systems require information to survive and proliferate. Heritable information in the genome encodes the structure and function of cellular macromolecules but this information remains fixed over time. In contrast, a cell must rapidly and continuously access, analyze, and respond to a wide range of continuously changing spatial and temporal information in the environment. We propose these two information dynamics are linked because the genes encode the structure of the macromolecules that form information conduits necessary for the dynamical interactions with the external environment. However, because the genome does not have the capacity to precisely locate the time and location of external signals, we propose the cell membrane is the site at which most external information is received and processed. In our model, an external signal is detected by gates on transmembrane ion channel and transmitted into the cytoplasm through ions that flow along pre-existing concentration gradients when the gate opens. The resulting cytoplasmic ion “puff” is localized in both time and space, thus producing spatial and temporal information. Small, localized signals in the cytoplasm are “processed” through alterations in the function and location of peripheral membrane proteins. Larger perturbations produce prolonged or spatially extensive changes in cytoplasmic ion concentrations that can be transmitted to other organelles via ion flows along elements of the cytoskeleton. An evolutionary constraint to the ever-increasing acquisition of environmental information is the cost of doing so. One solution to this trade-off is the evolution of information conduits that minimize signal loss during transmission. Since the structures of these conduits are encoded in the genome, evolution of macromolecular conduits that minimize signal loss is linked to and, in fact, governed by a universal principle, termed extreme physical information (EPI). Mathematical analysis of information dynamics based on the flow of ions through membrane channels and along wire-like cytoskeleton macromolecules fulfills the EPI principle. Thus, the empirically derived model of evolution by natural selection, although uniquely applicable to living systems, is theoretically grounded in a universal principle that can also be used to derive the laws of physics. Finally, if minimization of signal loss is a mechanism to overcome energy constraints, the model predicts increasing information and associated complexity are closely linked to increased efficiency of energy production or improved substrate acquisition.

## 1. Introduction

Living systems, uniquely in nature, use information to maintain a stable, highly ordered state while far from thermodynamic equilibrium [1,2]. The information necessary to maintain this tenuous existence can be divided into two forms. One is the well-studied genetic information that encodes the macromolecules necessary to form the physical structure of a cell. The other information [3] is about the cell’s environment, which is often spatially complex and temporally variable. A living system benefits from receiving this information to, for example, locate food sources or avoid predators but must also use energy and substrate to obtain and analyze this information. Thus, the optimal function of the living system requires maximally accurate information at minimum cost (i.e., expenditure of resources). This optimization requires the channel for signal transmission between the cell and its environment to have the property of minimum information loss, which is also a universal physical principle termed extreme physical information (EPI) [4].

Here, we propose that optimization of information channels between a cell and its environment promotes survival and proliferation. It is, therefore, promoted by evolutionary selection. However, it is also an expression of the EPI universal principle from which the laws of physics can be derived. Furthermore, minimization of information loss during transmission permits an increase in total system information and order, perhaps leading to increasing complexity in living systems over geologic time [5].

The dynamics governing information transmission in physical systems have been extensively investigated following the pioneering work of Shannon, Fisher, and others. Ideal information transmission requires that the receiver obtain all the information that was sent. In reality, there is almost an inevitable loss of information during the process of transmission, and optimization of communication remains a subject of great interest.

Traditionally in the physical sciences, information transmission has been used to describe how accurately a quantity defined on a continuum can be known from observation. However, over the recent two decades, another, completely different use of information has been developed through a principle of extreme physical information (EPI) [4] (see below). Rather than focusing on the particular value of an observable phenomenon, EPI seeks to determine the actual physical (probability) law governing that phenomenon [6]. Furthermore, while information observed is always less than that of the source, EPI postulates that the information loss is always an extremum, usually a minimum. The EPI principle has been used to derive virtually all of the laws of physics [7] (e.g., the Schrodinger equation [8]). More recently, empirically derived laws of biology (e.g., the Arrhenius equation [9] and Hodgkin-Huxley equations [10]) have been derived from the EPI principle.

Here, we extend this work by explicitly linking the fitness benefit of optimal (minimally lossy) information flow in living systems with the EPI principles that information loss during any physical transmission of any signal within the universe is always an extremum, usually a minimum. Thus, we propose the fundamental evolutionary laws of biology can be derived from universal first principles of the physical world.

How is information received in the cell membrane communicated to other components of the cell? Traditionally, communication is considered solely through molecular pathways that, following ligand binding to a membrane receptor, diffuse through the cytoplasm, often communicating to other diffusing proteins by acting as kinases [11]. However, within the EPI principle, these pathways are essentially switches and amplifiers [12] of the binding event (i.e., one ligand binding to a receptor may result in 10 or 100 s of diffusing phosphorylated proteins). This benefits the cell by transmitting information to multiple intracellular sites. However, this signal transmission is highly lossy as, for example, three-dimensional diffusion of messenger proteins in the cytoplasm greatly degrades information on the time and location (on the cell membrane) of ligand binding.

There are, however, other less well-recognized information conduits in the cell membrane in the form of ion channels. These dynamics were first observed in neurons as information as sequential opening of channel gates allowed flow of Na^+^ and K^+^ along previously established, energy-dependent transmembrane gradients produced a traveling depolarization wave along the membrane of an axon [13,14]. However, the large transmembrane ion gradients and gated membrane ion channels that produce the ion flows characterized by the Hodgkin-Huxley equation [15] are present in virtually all cells. This suggests transmembrane ion flows can serve as critical information conduits of non-neuronal cells.

An important property of the membrane ion information system is that it can resolve both spatial and temporal environmental perturbations since localized perturbation will produce a highly localized and transient ion “puff” [16] within the cytoplasm.

This allows rapid and spatially localized information processing as local changes in cytoplasmic ion concentrations can alter both the location and function of peripheral membrane proteins. Some environmental signals received at the membrane, because of their content, amplitude, or spatiotemporal frequency, may require a global (or ‘coordinated’) cellular response, such as increased energy production via mitochondria or changes in gene expression or translation. Here, the amplification process following ligand binding allows a broad distribution of signals to all critical subcellular organelles (e.g., nucleus, mitochondria, centrosome, etc.), permitting a coordinated response. Under some circumstances, this lossy information may be sufficient to elicit a necessary response. However, often, it may not. Clearly, when an environmental signal is time-dependent, and/or spatially localized in the membrane (e.g., a predator or prey in the case of a single cell eukaryote, or of location within a tissue in a multicellular organism), cell survival and/or optimal function may require additional input information, i.e., more complete temporal and spatial information.

**Figure 1:**
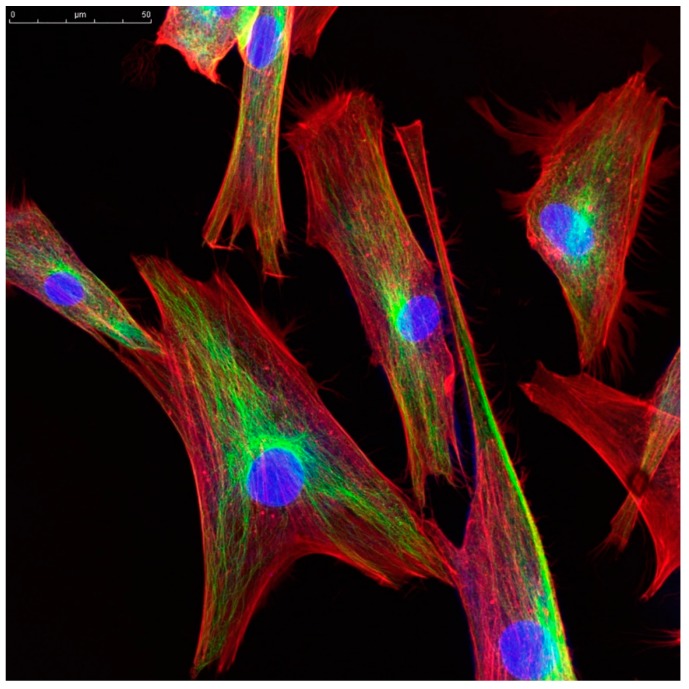
Immunohistochemistry stains from normal fibroblasts showing the distribution of microfilaments, which generally extend radially from the nuclear membrane to the cell membrane. We hypothesize the cytoskeleton, in addition to its biomechanical role, is a central factor in intracellular information transduction. The microfilaments are stained green with fluorescein isothiocynate-phalloidin. The nucleus (blue) is stained using 4′,6-diamidino-2-phenylindole (DAPI). While the cytoskeleton has a clear role in cellular shape and movement, multiple studies have demonstrated that both microfilaments and microtubules conduct ions. Note, the complex clustering for microtubules near the nucleus and the spoke-like connection of the nuclear membrane to the cytoplasm adjacent to the cell membrane allows rapid and spatially-defined information flow connecting ion-based information dynamics at the cell membrane with genomic information dynamics in the nucleus.

We propose that information encoded in local fluxes of the ion concentration in the cytoplasm adjacent to the cell membrane can be transmitted to other cellular organelles, or to other cells, by elements of the cellular cytoskeleton (Figure 1) [17]. Microtubules and microfilaments form organized, but highly variable, linear network in all eukaryotic cells. This is usually described as the cellular cytoskeleton and is clearly involved in cellular shape and movement as well. Both microfilaments and microtubules are highly dynamic and are constructed from subunits with highly charged surfaces. The microtubules are larger (about 30 nm in diameter), have a hollow core, and generally meet within the centrosome adjacent to the nucleus. The microfilaments are much thinner (about 3 nm in diameter) and form highly inter-connected networks. The microfilaments have a direct connection to the nuclear membrane via a large protein complex. Many empirical studies have found evidence for ion flow through the lumen of microtubules and along the highly charged surface of microfilaments [18,19,20,21,22,23,24]. Ion flow can result in the formation of an electromagnetic field, leading to additional dynamical interactions [24,25], including alterations in gene transcription [26] via the protein complex that links microfilaments to the nuclear membrane. Furthermore, these fibers are often arrayed in organized patterns, and are often observed to be oriented along the radius of the cell from the nuclear membrane to the cell membrane (see Figure 1). We hypothesize that these structures carry less lossy spatial and temporal information directly from the cell membrane to central cellular organelles, thus augmenting the signals carried by diffusing messenger proteins. The resulting signal could, for example, be processed in the microtubule or microfilament, resulting in local membrane movement or changes in shape that constitute a response to the environmental stimulus.

Here, we investigate the hypothesized ion-based information flow along elements of the cytoskeleton by applying the EPI principle. It is reasonable that, should such a system be present, evolutionary optimization over long periods of time would have selected for minimization of information loss identical to the EPI principle. Furthermore, we propose that the EPI principles can serve as the scientific foundation for the laws of evolution, which currently are derived solely from empirical observation.

## 2. Results

### 2.1. What Is Information?

The definition of information in a biological context can be controversial. Here, we view information in perhaps its simplest form, as a deviation from randomness in an observed effect suffering at least some anticipated level of unexpectedness [27,28,29]. For example, the difference between ion concentrations in extra-cellular and intra-cellular fluids represents a marked departure from randomness (which would favor equal concentrations). In fact, by generating intracellular ion concentrations compared to those in the extracellular space (which can be viewed as relatively constant), the cell creates a system in which the potential transmitted information is effectively very large (see below) and probably extremely valuable. In fact, the evolutionary value of the information can be roughly estimated by the cellular investment of resources—empirical studies have found that up to 40% of a cell’s energy budget is devoted to operating membrane pumps that maintain this transmembrane ion gradient [30].

Prior investigations have demonstrated that highly ion-specific transmembrane channels permit communication between the environment and the cell in the form of transmembrane ion flows. This occurs when the specialized gate (there are well over 100 different types of gates) is induced to open. Thus, the ion flow represents a signal regarding the nature of the perturbation as well as its time and place. In prior work [10], these information dynamics have been shown to be highly optimized consistent with the principle of minimum information loss.

The origin of information theory can be traced to a famous thought experiment (gedanken) by James Maxwell. He imagined two closed boxes of gas at identical temperatures connected by a single channel with a frictionless gate. A “demon” operated the gate and allowed only fast moving gas molecules pass in one direction and only slow moving molecules pass in the opposite direction. He proposed this would amount to a spontaneous flow of heat between structures initially at the same temperature – a violation of the First Law of Thermodynamics. Maxwell’s Demon remained a conundrum in the scientific world for over 40 years until it was noted that the Demon possessed and used information. This deep connection of information with thermodynamic properties such as entropy and energy as well as biological traits such as order and complexity emerges from this intellectual heritage (see Box 1).

In general, information transmission due to ion flow from outside to inside a cell membrane (CM) or linearly along fibers of the cytoskeleton is subject to two alternative principles of minimum information loss [17]: (i) *I – J* = minimum or $\left(\mathrm{ii}\right)\;KL_{1}-KL_{2}=\mathrm{minimum}.$ Subscripts 1 and 2 refer, respectively, to the levels of information outside of and inside the CM. These can be present, e.g., at multimolecular connections between the cytoskeleton and the nuclear membrane. In principle, (i) *I* and *J* are, respectively, levels of temporal Fisher information [4,27,31] over the continuous, total time interval (*0*,*T*) of flow within the cell membrane. Or, in principle, $\left(\mathrm{ii}\right)\;KL_{1}$ and $KL_{2}$ are levels of Kullback-Leibler divergence [9,10,27,28], or relative entropy, over the time interval (*0,T).* Then, principle (ii) becomes the difference between Kullback-Leibler divergences for ion flow values entering and leaving the cell membrane.

As to whether the appropriate principle is of type (i) or (ii) is according to how finely structured the neurons are. As shown below, in systems with a true fine structure (order of 1–5 nm), information, *I*, is the ion’s level of Fisher information. In principle (i), depending on the case, *J* could be the sum of all ion physical constraint conditions (normalization, e.g., known mean times within the system, etc.) affecting *I.* Or, *J* could be the information as represented in a conjugate space to *t,* such as energy-momentum in quantum-relativistic problems.

**Figure 2:**
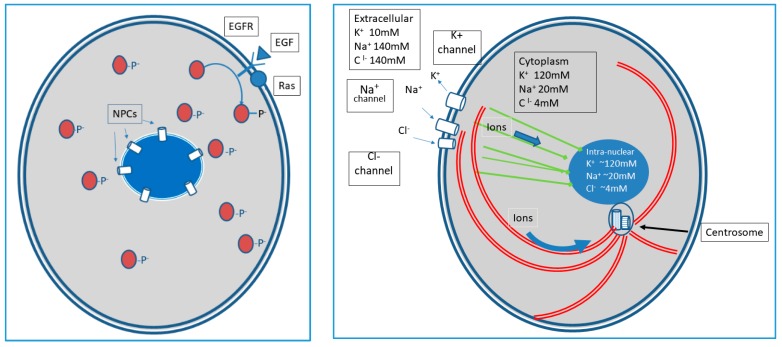
A summary of the proposed intracellular information dynamics. In the left panel, information in the form of a ligand binds to a membrane receptor. The message is amplified by phophorylating multiple copies of the pathway protein (blue curved line). However, because the phosphorylated messenger protein must diffuse in three dimensions and then pass through nuclear pore complexes (NPCs), the location on the membrane and the time at which the ligand arrived is degraded. In the right panel, the green lines represent microfilaments (see Figure 1) and the red, curved lines are the microtubules. We propose a theoretical model of intracellular dynamics based on ion flow. About 30% of a cell’s energy budget is used to pump ions across the cell membrane to establish large gradients. Most cells maintain large numbers of membrane channels specific for each ion (Na^+^, Cl^−^, K^+^, Mg^2^^+^, and Ca^2+^). The channels have specialized gates that respond to specific environmental perturbations (hundreds of different kinds of gates are encoded in the genome). This produces intracellular information flow as ions flow through the open membrane along concentration gradients. These local and brief changes in cytoplasmic ion concentrations generate gradients (between the peripheral cytoplasm and nucleus) along microfilaments (green—see Figure 1). Microfilaments are excellent conductors allowing ions to flow rapidly along the fibers to the nuclear membrane adhesion proteins that can alter chromosomal localization and gene expression (see curved arrow in right panel). Similarly, ions can flow along microtubules (straight arrow in right panel) to and from the centrosome, which can serve as an integrator of course-grained information throughout the cell and communicate with the adjacent “central” structure including the nucleus, endoplasmic reticulum, and mitochondria. Note the similarity of the membrane ion dynamics to those in the neuron axon during wave propagation and framed through the Hodgkin–Huxley equations. This model proposes the ion flows that conduct these traveling depolarization waves in neurons are a specialized form of membrane information dynamics found in all eukaryotes. In fact, the Hodgkin-Huxley equation can be derived from the Information Theory equations in the text.

The application of these information dynamics to individual cells is demonstrated in Figure 1 and Figure 2. Here we view the classical macromolecular pathways as signal amplifiers. Our focus is primarily on recently described non-genomic information reception, processing, and response mechanisms that are primarily governed by flows of ions between the intra- and extra-cellular spaces across the cell membrane and intracellular ion flows along elements of the cytoskeleton.

Principle (i), operating on the finest level of cellular structure, has been called EPI [7] (extreme physical information). On this scale, the principle (i), of *I – J* = minimum, often gives rise to quantum effects, such as the Schrodinger wave equation (here, in particular, *J* is the mean kinetic energy). The principle (i) is then explicitly one of temporally minimum Fisher information loss. This assumes decoherence effects to be negligible.

Alternatively, for ions flowing through coarser-grained microtubules (order of 25–100 nm), the EPI principle becomes one of (ii) minimum loss of Shannon information (ii) ΔS ≡ $KL_{1}-KL_{2}=minimum$. This is a non-quantum, coarser-grained theory. In fact, principle (ii) directly derives as a coarser-grained version of principle (i). As a verification, in the case of ion flow through the Giant Crab neuron, one solution of type (ii) of coarser ion flow is found to obey the classical Hodgkin–Huxley equations [15].

### 2.2. Fisher Information I

All information forms used in this paper ultimately arise out of Fisher information. By definition, this obeys [4,6,32]:

(1) $$I=4\int dt\;{\left[\frac{da}{dt}\right]}^{2},\;a=a\left(t\right)\equiv \sqrt{\;p\left(t\right)}$$

where p=p(t) is the probability density on position *t* for the ion and a(t) is defined to be its real (for simplicity here) amplitude, $\sqrt{p\left(t\right)}$. All integrals are over a fixed time interval, 0 ≤t≤T.

For now, we notice that the form of Equation (1) is also that of a Lagrangian, L, in integral ∫dtL, and this is conventionally varied as δ∫dtL=0 to derive the quantum mechanics obeyed by the amplitude law, a(t), in scenarios of fine structure, 1–5 nm, or, alternatively, by classical flow of p(t) in a coarser structure in the range 25–100 nm. The emphasis here is on the latter (classical domain) behavior.

The information, *I*, defined by Equation (1) is also conventionally used to determine, by the relation:

(2) $$e_{min}^{2}=1/I$$

The minimum possible mean-squared error, $e_{min}^{2}$, of any estimate of the true time, *t_0 based_*, is based on its repeated observation *N* times. Thus, *I* measures how well a quantity defined on the continuum (here of time values *t*) can be known out of observation (notice that in Equation (2), the larger the information, *I*, is, the smaller the *rms* error, *e_min_*, as one would expect of an information measure). This has been the basis for usual past uses of Fisher information, *I.*

By comparison, over the recent two decades, another completely different use of information, *I*, has arisen. Its aim is not to merely measure particular values *t_n_* of an observable phenomenon, as above, but rather to estimate the actual probability law, *p*(*t*), governing *t* in the unknown phenomenon (of physics, econophysics, biology, cancer growth, chemistry, etc.).

### 2.3. Its Physical Manifestation J, EPI Principle

This is by using a principle of extreme physical information [4,33,34]:

*I – J* = minimum (3)

through variation of *p*(*t*). Although both *I* and *J* are ‘informations’, they differ basically. From the factor ${\left(\frac{da}{dt}\right)}^{2}\;$in Equation (1), information *I* governs the amount of ‘slope’ or ‘roughness’ in both the probability law, p(t), and its amplitude law, a(t). Also, by Equation (2), *I* governs how accurately an unknown coordinate *t* can be known. The other quantity, *J*, in Equation (3) defines the meaning of the information, *I*, as a physical quantity. Their difference, *I – J*, is called the ‘physical information,’ so Equation (3) expresses a principle of extreme physical information (EPI). Since it is always a minimum value principle, Equation (3) (or principle (i)) represents a scenario of minimum lost temporal Fisher. What does this mean?

Quantity *I – J* is always convex, so it defines a minimum value when varied mathematically. Such minimization means that I≈J, i.e., the theoretical information tends to equal its physical manifestation (in fact, in quantum scenarios, I=J, meaning the entire physical manifestation, J, of information *I* (here the energy) goes into forming the observable information, I.

The EPI principle (3) provides the solution for amplitudes a=a(t) in ion flow on the fine structured (quantum) time scale. The principle has been used to derive^7^ most laws of textbook physics and some laws of biology. The latter include the mass growth law, $m\left(t\right)=m\left(0\right)t^{\emptyset }$, for early growth stages of breast cancer [35,36], where the constant, ∅=1.618034…, is the Fibonacci number so often found to arise in biological growth effects. That it governs cancer mass growth is, then, consistent with such past usage.

### 2.4. Transition to Principle of Minimum Kullback–Leibler (KL) Divergence

We saw that the Fisher information-based EPI principle (3) is directly applicable to problems of unknown ion rate functions, p(t) or a(t), on the continuum of *t,* typified by spatial observations on the nanometer (fine) scale, 1–10 nm. For this scale of problem, information *I* was found to be Fisher’s, given by Equation (1). However, ions can move along larger conduits, such as the microtubule (~30 nm diameter). This is a coarse-grained problem. To find the ‘coarse-grained’ rates, p(t), q(t), we examine the transition from the fine-scaled principle (3) to the corresponding coarse-grained problem.

We start by using identity, $p\left(t\right)=a^{2}\left(t\right)$, in Equation (1) to express it in terms of the probabilities, p(t):

(4) $$\;I=\int dt\frac{{\left(dp/dt\right)}^{2}}{p}$$

For application of the EPI principle (3) to this discrete problem, we need the form of information (4) where differentials, dt, are regarded as small but finite changes, Δ*t.* This is easily found to give, as an approximation, [4]:

(5) $$I=\;2/\left(\Delta t^{2}\right)\int_{0}^{\infty }dtp_{i}\left(t\right)\ln(p_{i}\left(t\right)/q_{i\;}\left(t\right))\;=minimum$$

for i= 1,…,N. Factor 2/(Δt^2^) in Equation (5) shows that observing the time with a finer (smaller) ‘grain size’, Δt gives greater information, I, in a cell structure, such as a tubule. This is intuitively correct. Also, the information integral (4) thereby becomes proportional to the Kullback–Leibler (K-L) ‘divergence’ (right-hand integral in Equation (5)) between the ingoing, $p_{i}\left(t\right)$, and outgoing flows, $q_{i}\left(t\right),$ respectively. This K-L divergence is also known^3^ to be the Shannon information [27] for the membrane regarded as an information channel. By Equation (5), it is, then, explicitly, minimized in this coarse-grained scenario. It is therefore identically the loss in Shannon information during the flow from outside to inside the tubule. Thus, although Equation (5) indicates that Shannon information can only be lost in this transition, the loss must be a minimum. This is central to the information-based approach here.

Why? Dynamically, the ion signal travels from its information source to the receiver through an environment that will add thermal random motion (noise). It therefore can only lose information en route. However, since this loss *I – J =* minimum, it means that the received level of information is actually the maximum possible over all such environments.

Thus, in summary, both principles (i) and (ii) define scenarios of minimum loss, or equivalently maximum gain, of information. These are of different types: Fisher information in (i), and Shannon information in (ii).

### 2.5. Insertion of Prior Knowledge

The minimum in Equation (3) is to be obtained in the presence of prior knowledge, *J*, about the trajectories. These must, e.g., obey normalization. However, the key prior knowledge was found by Hodgkin and Huxley to be the mean times, $\tau_{i},\;i=1,\ldots ,N$, for ions in the system. The minimum value in Equation (5) is also constrained by this knowledge. Using these as additive Lagrange constraints, *J*, on the minimization of *I,* Equation (5) becomes one of constrained K-L divergence, *KL* [10]:

(6) $$\begin{array}{c} I-J=\int_{0}^{\infty }dtp_{i}\left(t\right)\ln(p_{i}\left(t\right)/q_{i\;}\left(t\right))+\lambda_{1}\int dt\left[p_{i}\left(t\right)-1\right]+\lambda_{2}\int dt\left[q_{i}\left(t\right)-1\right]\\ \;+\lambda_{3}\left[\int dttp_{i}\left(t\right)-\tau_{i}\right]=min \end{array}$$

As we saw, this principle maximizes information dynamics through transmembrane flow of ions. The first right-hand term is the *KL* divergence in Equation (5). The terms in $\lambda_{1}$ and $\lambda_{2}\;$express the normalization of probability densities, $p_{i}\left(t\right)$ and $q_{i}\left(t\right)$. The term in$\;\lambda_{3}$ expresses knowledge of mean times, $\tau_{i}.\;$Equation (6) is of the same general form as the EPI principle (3) and, hence, is called the *KL*_min_ principle. It has been found that this *KL*_min_ principle derives the correct equations governing the transmembrane ion flows, $p_{i}\left(t\right),\;i=1,\ldots ,N$, that produce traveling depolarization waves in a neuronal axon, i.e., the Hodgkin–Huxley equations [10,15].

## 3. Discussion

### 3.1. Implication for Natural Selection

In evolution, the fitness of an individual is determined by the interactions of its phenotype with local environmental properties that act as selection forces. However, the environment is rarely constant and so, as an organism migrates from one region to another or remains fixed in space but is subjected to weather- and seasonal-related changes, its fitness will similarly vary. Living systems, uniquely in nature, can access information in their environment to adjust their phenotypic properties or behavior to maintain fitness.

Generally, information dynamics in biological systems are viewed solely in the context of genetics. In evolution, the role of the genome is clearly that of the mechanism of inheritance. That is, the genome determines the structure of the organism. This information is critical in determining the fitness of the cell by encoding phenotypic plasticity and the mechanism by which environmental information is obtained. However, is the genome the command center of the brain that detects, processes, and responds to all environmental perturbations? Here, the limitations of diffusion-based signal transmission by classic transduction seem clear—the nucleus and genome cannot resolve spatial or short term temporal information within the environment. As discussed above, unlike the nucleus, the cell membrane, as the interface between the cell and its environment, must be the site at which external information is received, processed, and communicated to other organelles, including the nucleus.

As demonstrated above, information dynamics related to ion flows both across the membrane and along elements of the cytoskeleton can optimally contribute to cellular fitness when signal transmission is constrained to minimize loss. That is, by allowing the cell to gain maximal amounts of information from the environment and minimal cost, it can rapidly and accurately detect as well as quickly deploy an integrated cellular response to environmental threats or opportunities. This link between fitness and information permits an explicit connection between the principle of evolution and the universal principle of extreme physical information that has previously focused primarily on physical science application. That is, since Darwin [37], it has been clearly recognized that living systems are subject to the principle of natural selection, or survival of the ‘fittest’. Clearly, fitness is increased by optimal information flow within a living system. This optimization, in turn, must obey physical information-based principles as described above. Thus, the concepts of optimal fitness and minimum information loss can be theoretically linked [38].

Box 1 **Mathematical framing of information theory and the principle that information flow in nature obeys a universal law of minimum loss (Extreme Physical Information [EPI]). The applicable mathematical applied to the information dynamics is spatially dependent and divided into coarse grained or fine grained. Both apply to living systems and the latter permits quantum phenomenon in biology. Here, we propose EPI is a universal principle that gives rise to evolutionary dynamics.**

All mathematics describing information dynamics are derived from the Fisher equation, which describes the transmission of the probability law, which defines the source of information:

(7) $$I=4\int dt\;{\left[\frac{da}{dt}\right]}^{2},\;a=a\left(t\right)\equiv \sqrt{\;p\left(t\right)}$$

where a represents *p(t)* is the probability density of the observed phenomenon and *a(t)* is the amplitude, $\sqrt{p\left(t\right)}$, over some fixed time period.

The principle of extreme physical information can be simply stated as:

*I* – *J* = *minimum* (8)

where *I* and *J* are both informations, with *J* representing the probability functions governing the observed phenomenon and *I* is the information received by the observer. That is, *I* governs how accurately an unknown coordinate, *t*, can be known. The other quantity, *J*, in Equation (8) defines the meaning of the information, *I*, as a physical quantity. Their difference, *I – J*, is called the ‘physical information’, so Equation (8) expresses a principle of extreme physical information (EPI). In effect, Equations (7) and (8) simply state the universal principle that information transmission in nature invariably occurs with a minimum information loss. Or, alternatively stated, that a maximum amount of information is conveyed.

In the physical sciences, information can be conveyed in very small scales (quantum level) or macroscopic scales governed by Newtonian dynamics. Similarly, the mathematics of information transmission is different at each spatial scale. Information of small (fine grained) continuous processes is defined as Fisher information (Equation (7)). However, on larger scales, the probability distribution will describe discrete (rather than continuous) structures. In this setting, the information equation is:

(9) $$I=2/\left(\Delta t^{2}\right)\int_{0}^{\infty }dtp_{i}\left(t\right)\ln(p_{i}\left(t\right)/q_{i\;}\left(t\right))\;=minimum$$

Note this still represents a minimization of information loss as in Equation (8). It is simply a modification of the Fisher equation to describe the coarse-grained phenomena. This equation is termed the Kullback–Leibler (K-L) information. The amount of information lost is termed the K-L divergence. This can be visualized spatially as a lock and key. The more precisely they fit, the lower the K-L divergence.

In cells, microfilaments, with a diameter of 3 to 5 nm, are fine-grained structures. In fact it is theoretically possible that quantum effects can occur in microfilaments. In contrast, microtubules are coarse grained and thus described by the K-L divergence. Another interesting coarse-grained biological structure is the axon of a neuron. It is illustrative that the K-L divergence can be used to derive the Hodgkin–Huxley equation of the propagating wave in neurons from first principles (the original equation was empirically derived base on curve fitting to experimental observations).

Furthermore, if the EPI principle is obeyed on the cell micro level, it will give rise to living systems exhibiting high levels of fitness on the macro level. This bridging of spatial and temporal scales arises in the following way. Denote the information flow to be $p_{i}\left(t\right),\;i=1,\ldots ,N$ for a population and $p_{j}\left(t\right),\;i=1,\ldots ,N\;$for population. Inevitably, these dynamics will be more favorable for one group than the other. Such would be the case, e.g., if the amount of substrate utilized to attain the optimally high rates {i} are lower than the group {j}. Then, at some time, $t=t_{0}$ rate$\;p_{i}\left(t\right)>p_{j}\left(t\right).$

This advantage may initially be quite small, but the population of organisms with a higher survival rate will increase with time, usually exponentially. Thus, with time, the disparity between the two alternatives becomes ever larger, and in the end (after a large number of generations), only one line of development {i} survives. In this way, the very process of evolution keeps its “subject” organisms optimal for survival.

However, life overall is robust, so that some accommodation might simultaneously be made to attain higher rates for group {j}. This would be at the expense of the optimum level of substrate for solutions {i}. With levels {i} so reduced sufficiently, the increased available level of substrate probably could elevate levels {j} to acceptable values. An example is the diffusing protein pathways that carry information about ligand binding. These serve as amplifiers of one component of the information (the ligand binding event) but can tolerate loss of information about the time and location at which the ligand is bound to a receptor. In our model, the information lost in the macromolecular pathways is regained through the ion conduction dynamics that obey the EPI principle. In this way, organisms may use a mixture of signal transmission conduits when the overall effect optimizes communication within a system containing multiple elements [39].

Finally, we note that a clear pattern in evolution in the history of life on earth is toward greater complexity as demonstrated by prokaryotes, eukaryotes, and increasingly larger and more sophisticated multicellular organisms. The generally increasing relationship of information and complexity has been well-documented [40]. Minimizing information loss through evolution also permits maximum gain, particularly when energy becomes more abundant (i.e., acquisition of mitochondria in the transition from prokaryotes to eukaryotes).

### 3.2. Information, Order, Fitness, Evolution and Universal Principles in Nature

Here, we distinguished heritable information in the genome from the continuous information flow from the environment to a living system, such as a cell. The latter determines the physical structure of the cell, including the properties of its information channels. However, spatially explicit environmental information continuously over time is detected and processed primarily in and around the cell membrane. In this model, the genome is a source of macromolecules when needed but not the central command center of the cell as it is commonly viewed.

We propose that Darwin’s empirically based theory of evolution by natural selection can also be derived theoretically through a variational principle of biophysics that depends upon the concept of information flow. Here, the information flows from the environment to the cell and must include spatial and temporal information with, while Fisher information loss is minimized in fine-grained channels, such as microfilaments. However, “minimum loss” also means, in a positive sense, maximum gain. Here, the gain is the fitness of the individual but it also includes a generalized increase in organismal information, a necessary condition for the increase in complexity from prokaryotes to eukaryotes to progressive larger and more sophisticated multicellular organisms over geologic time [41].

Here, we showed that the particular form of information depends upon the state of granularity of the medium through which information flows. That is, loss of Kullback–Liebler or Shannon information is minimized in coarse-grained channels, such as microtubules, while Fisher information loss is minimized in fine-grained channels, such as microfilaments. However, “minimum loss” also means, in a positive sense, maximum gain. Here, the gain is the fitness of the individual but it also includes a generalized increase in organismal information, a necessary condition for the increase in complexity from prokaryotes to eukaryotes to progressive larger and more sophisticated multicellular organisms over geologic time.
